# Radiographic Evaluation of the Gap between Cemented Post and Remaining Gutta-Percha in Endodontically Treated Teeth Performed by Undergraduate Students: A Retrospective Cross-Sectional Study

**DOI:** 10.3390/medicina59030502

**Published:** 2023-03-03

**Authors:** Hina Naim, Manawar Ahmad, Abrar A. Ageeli, Raghad K. Abuarab, Mohammed E. Sayed, Harisha Dewan, Hitesh Chohan, Abdullah Hasan Alshehri, Mohammed Hussain Dafer Al Wadei, Saeed M. Alqahtani, Shaikh Mohammad Abdul Feroz, Amit Porwal, Ahid Amer Alshahrani

**Affiliations:** 1 Department of Prosthetic Dental Sciences, College of Dentistry, Jazan University, Jazan 45142, Saudi Arabia; 2 Specialty Dental Center, Ministry of Health, Jazan 45142, Saudi Arabia; 3 Prosthodontic Department, Ministry of Health, King Fahad General Hospital Jeddah, Jeddah 23454, Saudi Arabia; 4 Department of Restorative Dental Sciences, College of Dentistry, Jazan University, Jazan 45142, Saudi Arabia; 5 Department of Prosthetic Dentistry, College of Dentistry, King Khalid University, Abha 62529, Saudi Arabia; 6 Department of Restorative Dental Science, College of Dentistry, King Khalid University, Abha 62529, Saudi Arabia; 7 Department of Dental Technology, Applied Medical Sciences, King Khalid University, Abha 61413, Saudi Arabia

**Keywords:** post and core, post gap, gutta-percha, endodontically treated teeth

## Abstract

The coronal seal in root canal-treated teeth may be compromised depending on the accuracy of post space preparation and post cementation along with remaining gutta-percha. Root canal treatment can be compromised by endotoxins released by the coronal bacteria as a result of microleakage. The study was conducted by undergraduate students to measure the gap between the cemented post and residual gutta-percha. In total, 217 endodontically treated teeth were evaluated with intraoral peri-apical radiographs. Based on the intraoral periapical radiographic examination in the CS-R4 program, Group I had no gap, Group II had a gap of >0 to 2 mm, and Group III had a gap of more than 2 mm between the end of the cemented post and the remaining gutta-percha. In total, 40% (*n* = 87) of the teeth had no gap, 59% (127) had a gap of >0 to 2 mm, and 1% (*n* = 3) had a gap of more than 2 mm between the cemented post end and remaining gutta. Chi square test revealed a significant difference in the gap between the post and remaining gutta-percha between males and females students (*p* < 0.001). In terms of the gap between the cemented post end and the residual gutta-percha, the root canal treated teeth with post and core by undergraduates were clinically acceptable.

## 1. Introduction

The majority of teeth requiring endodontic treatment have significant damage, which necessitates a post and core restoration before the final restoration. There are several factors that determine the success of endodontic treatment such as preoperative apical status, obturation techniques, materials, and the quality of coronal seal [1,2,3]. The fracture resistance of an endodontically treated tooth with post is influenced by post length, post diameter, remaining dentin, post material, post adaptability, post design, cement, core material, biocompatibility of the post material and load experienced by the restored tooth [4]. During post and core treatment, gutta-percha material may not provide an adequate seal in the root canal, compromising the coronal seal. Endotoxins released by coronal bacteria as a result of microleakage can lead to endodontic therapy failure [5]. Immediately following root canal therapy, it is important to seal the root canal and minimize the leakage of oral fluids and bacteria into the peri radicular areas to improve the success of root canal treated teeth [6]. Post space preparations should be extended such that 3 to 5 mm of gutta-percha remain at the apex of the tooth [7].

Microleakage can be affected by the methods of root canal therapy, the quality of root canal filling, timing of post space preparation and post cementation employed [8,9,10]. In the study conducted by Fan et al., delayed preparation caused more coronal leakage than an immediate preparation [11]. Posts cemented with dentin bonding resin cements exhibit less microleakage than those cemented with non-dentin bonding cements [12]. It might be better to restore the tooth immediately with a prefabricated post and composite core system than placing a temporary post and then a cast post and core [13]. 

After the cementation of post in root canal of endodontically treated teeth, there is usually a gap between the apical end of the post and the most coronal portion of gutta-percha. It has been claimed that the distance between the post and the remaining root canal filling is another crucial determining factor in the inclusion of microorganisms after post restorations. However, only a small number of studies have looked at how this gap affects the success of teeth that have had endodontic treatment. Since there is a lack of literature in this area, it would be useful to determine the success of endodontic treatment based on the gap between the post and the root canal filling. This cross-sectional study was aimed at finding the gap between the post end and remaining gutta-percha in the root canal of endodontically treated teeth by the undergraduate students and to evaluate if undergraduate students were sufficiently trained for doing post and core treatment on patients after doing preclinical laboratory training. The null hypothesis tested was that there would be a gap between the cemented post end and the remaining gutta-percha in the root canal-treated teeth by the undergraduate students.

## 2. Materials and Methods

A cross-sectional retrospective research design was used in the study. A total of 97 patients were included in the study. These patients were treated with post and core after the endodontic treatment by the final year undergraduate students. The same students were previously trained to perform the post and core procedures as part of their Preclinical Training in Fixed Prosthodontics at the College of Dentistry, Jazan University, Saudi Arabia. An ethical approval under reference no. REC42/1/051 was taken prior to the start of the study. During the endodontic treatment procedure, teeth were first isolated with rubber dam. Dental caries and old fillings were removed, and a standard endodontic access was established. With the help of an endodontic explorer, the pulp chamber was examined, and canal orifices were observed. K-files (Dentsply Sirona) were used to explore the canals. A radiograph with K-files was used to measure and confirm the working length of each root canal. Following that, the root canals were cleaned and shaped using the crown-down method. During the procedure, 17% EDTA gel was used as a lubricant and 5.25% sodium hypochlorite solution was used as an irrigant. The final irrigation method used normal saline. Calcium hydroxide was used as intracanal medication and zinc oxide eugenol was used as a sealer. The canals were cleaned with regular saline, dried with absorbent paper points, and obturated using the warm gutta-percha with lateral condensation technique. After the root canal procedure was finished, a composite resin (Z350 3M, USA) was used to restore the tooth. To evaluate the effectiveness of the obturation, a final periapical radiograph was taken.

During the post and core treatment of endodontically treated teeth, post space was prepared using Gates Glidden drills and peeso reamers (MANI, Japan size #1 to #4) depending on the diameter of the canal and an endodontic hand instrument to accept the post. The canal was prepared in a manner that ensured 5 mm of gutta-percha to maintain the periapical seal. The length and diameter of prefabricated reinforced glass fiber post (3M RelyX, St. Paul, MN, USA) was selected according to the shape and diameter of the root canal. The final shape of post space was prepared with the corresponding size universal drill (white for diameter 1.10 mm; yellow for diameter 1.30 mm; red for diameter 1.60 mm and blue for diameter 1.90 mm) supplied by the post manufacturing company. The apical seal and post-space preparation were evaluated with intra oral radiograph. The post space was cleaned and dried with paper point. The post was inserted and luted with self-adhesive resin cement (3M RelyX Unicem 2 Automix, St. Paul, MN, USA) under isolation with a rubber dam. The automix syringe, together with the endo tip, was used to apply the cement directly into the root canal, reducing the chance of air pockets and voids. Custom metal posts were fabricated using an indirect method. A post space impression was made using pattern resin (GC America, Pattern resin) and plastic speedy post after the separating media had been applied to the post space that had been prepared. The core portion of the crown was shaped with pattern resin at the same time taking into the consideration of the shape of future abutment teeth. These post and core pattern resins were sprued, invested, and cast following the manufacturer’s guidelines. The retrieved castings were tried in the patient’s mouth, and the final finishing was performed intraorally. Zinc phosphate cement (Primedent, St. Paul, MN, USA) was used to cement the metal cast posts. 

Sample size calculation was performed using the R statistical package, version 3.3.1 (R Core Team 2016; R Foundation for Statistical Computing, Vienna, Austria). One-way analysis of variance power calculation for more than two groups was used to detect the proper sample size. The results showed that, at a power of 90% and a two-sided significance level of 5%; a total sample size of 210 endodontically treated teeth would be adequate to reject the null hypothesis that among all the three groups there is no gap between the post end and remaining gutta-percha. 

Inclusion criteria for the study were:

1. Only patients treated for root canal treatment and post and core by the undergraduate students.
2. Only patient records containing digital periapical radiographs taken immediately after the post and core treatment present.

Exclusion criteria for the study were:

1. Patients treated by the specialists.
2. Cases in which there were additional factors that could influence the outcome of the root-canal treatment, such as broken instruments in the root canal, over- or under-extension of the root canal filling, or root fracture, were excluded.

**Figure 1:**
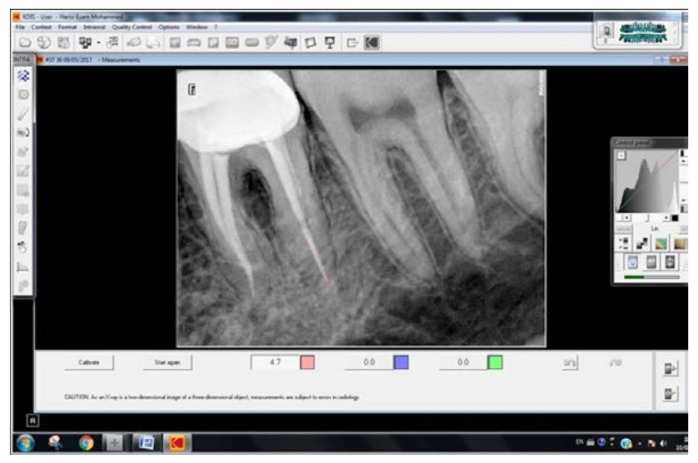
A peri-apical radiograph showing the measurement of gap between the cemented post and remaining gutta-percha filling using the CS-R4 program.

**Figure 2:**
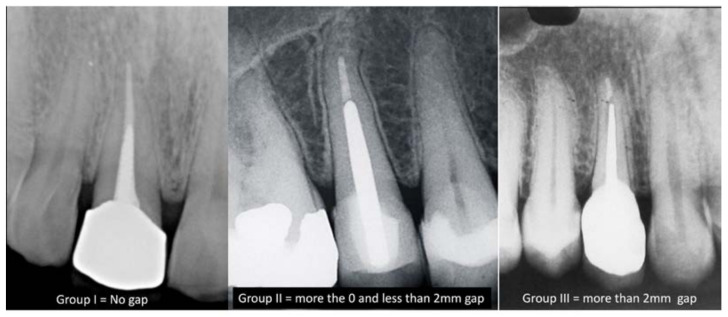
Peri-apical radiographs showing three different groups (Group I, II and III) for gap between the cemented post and the remaining gutta-percha.

A total of 217 endodontically restored teeth with post and core treated with prefabricated glass fiber post and custom cast post were included in the study. The intraoral peri-apical radiographs of the cemented posts were made using paralleling technique. The radiographs were examined in the CS-R4 program (CSR4 plus Practice Management Software version 4, Carestream Dental LLC, Atlanta, GA, USA) (Figure 1). According to the gap present between the apical tip of the cemented post and remaining gutta-percha in the radiographs, the cases were divided into three groups [14] (Figure 2):

Group I: no gap between the cemented post and the gutta-percha.
Group II: a gap of more than 0 but less than 2 mm between the gutta-percha and the post.
Group III: a gap of more than 2 mm between the gutta-percha and the post.
Additionally, the same cases were divided into three different groups to evaluate the amount of remaining gutta-percha after post and core treatment [15,16]:
Group IV: less than 3 mm of gutta-percha remaining.
Group V: 3 to 5 mm of gutta-percha remaining.
Group IV: more than 5 mm of gutta-percha remaining.

All radiographs were assessed independently by two examiners. Before performing the radiographic evaluation, both of the examiners were calibrated. The study methodology was explained to the examiners during the calibration phase. To minimize discrepant results, the examiners also familiarized themselves with the scores they should attribute to the radiographic images and the established evaluation method for the study. Initially fifty teeth were evaluated to calibrate both examiners and inter examiner agreement was detected by Cohen’s kappa (kappa 0.91). To provide optimal radiographic image quality and radiographic contrast, the surrounding light was controlled by darkening the room. When disagreement occurred between 2 examiners, a third observer, a prosthodontist with 10 years of clinical experience, was asked to make a decision. Each measurement was taken three times by one expert investigator after software calibration for linear measurement. To minimize the intra-observer error, the average of three values were obtained. The data were collected between December 2021 and June 2022. All the qualitative data were assembled into different groups according to the gap present between the post and remining gutta-percha. These data were also grouped according to the post- and core-treated teeth among the male and female students. The data were analyzed using the Microsoft Excel software package (version 2016) and summarized in tabulated form. A chi-square test of independence was implemented for all the findings using SPSS software (version 21, SPSS, Chicago, IL, USA) with the level of significance set at *p* = 0.05.

## 3. Results

Among the 97 patients, a total of 217 endodontically restored teeth with post and core were enrolled. Most of the patients had multiple post- and core-treated teeth. Of the selected teeth, there were 105 post- and core-treated teeth where the treatment was performed by male students and 112 post- and core-treated teeth where the treatment was performed by female students. The randomly selected eligible patients recruited in the study underwent the intra-examiner (i.e., the same examiner making two independent measurements on the same radiograph at recruitment and at final measurement of sample) and the between-examiner or inter-examiner (i.e., two examiners making two independent measurements on the same radiograph) evaluation at the same time.

**Figure 3:**
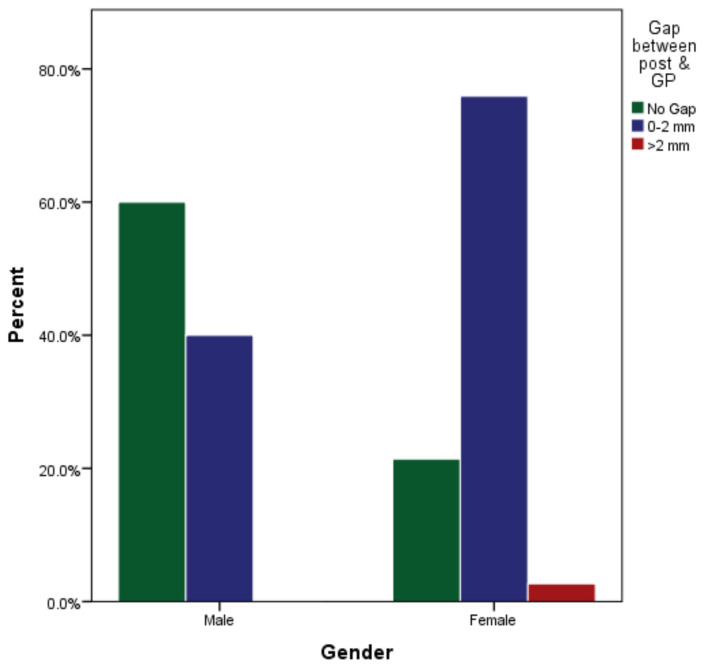
Gap between post and remaining gutta-percha among male and female students.

Of the 217 endodontically treated teeth, 87 (40%) had no gap between post and core restorations, 127 (59%) had a gap of >0 to 2 mm, and 3 (2%) had a gap of more than 2 mm (Table 1). On 105 endodontically treated teeth restored with post and core by male undergraduate students, 63 (60%) had no gap, 42 (40%) had a gap of >0 to 2 mm, and none had a gap over 2 mm (Figure 3). Of the 112 post and core teeth treated by female undergraduate students, 24 (21%) had no gap, 85 (76%) had a gap of >0 to 2 mm and 3 (3%) had a gap of more than 2 mm (Table 2). There was a significant difference in the gap between the post and remaining gutta-percha between male and female students (*p* < 0.001).

**Figure 4:**
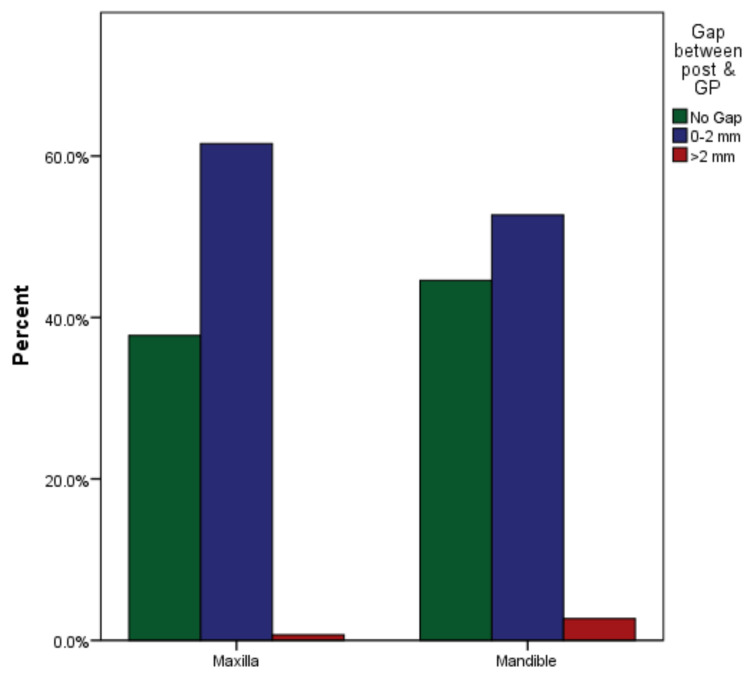
Gap between post and remaining gutta-percha in maxillary and mandibular arches.

Out of 143 teeth, 54 (38%) were restored without any gaps, 88 (62%) had gap between 0 and 2 mm, and one tooth (0.7%) with more than 2 mm gap in maxillary arch (Table 1). Of the 74 teeth with post and core in the mandibular arch, 33 (45%) were restored without a gap, 39 (53%) teeth had a gap of >0 to 2 mm and 2 (2.7%) teeth had more than 2 mm gap (Figure 4). Between the maxillary and mandibular arch, no significant gaps were found between the end of the cemented post and the remaining gutta-percha (*p* = 0.268).

**Figure 5:**
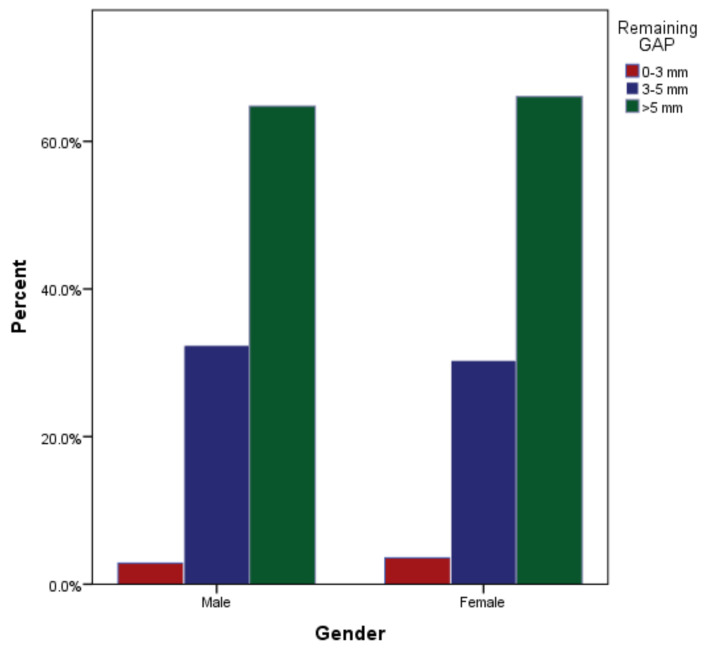
Remaining gutta-percha after post cementation among male and female students.

**Figure 6:**
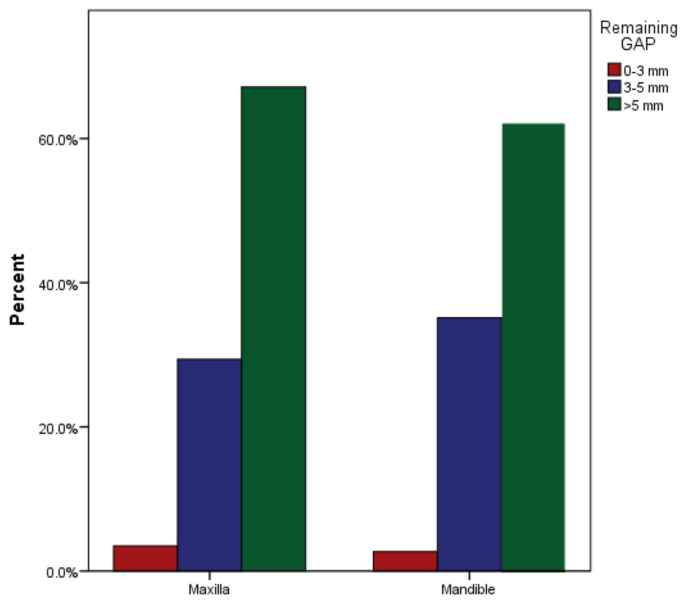
Remaining gutta-percha in maxillary and mandibular arches after post cementation.

As far as the remaining gutta-percha is concerned, the majority of cases (*n* = 68, 65% for males and *n* = 74, 66% for females) with 142 (65%) had more than 5 mm of gutta-percha apical to the posts (Table 3). The gutta-percha apical to the post end was 3 to 5 mm in 34 (32%) and 34 (30%) of the males and females, respectively. Seven of the total cases (3%) had less than 3 mm of gutta-percha remaining (males: 3.3%; and females: 4.4%). The difference was not clinically significant between males and females (*p* = 0.918, Figure 5). As far as the remaining gutta-percha at the cemented post apical section is concerned, the majority of cases (*n* = 96, 67% for maxillary arches, *n* = 46, 62% for mandibular arches) showed more than 5 mm of gutta-percha apical to the cemented post end (Table 3). It was found that there were 42 (29%) teeth endodontically treated in the maxillary arch and 26 (35%) in the mandibular arch with 3 to 5 mm of remaining gutta-percha apical to the cemented post (Table 4). Only seven (3%) of the total cases showed less than 3 mm of remaining gutta-percha (*n* = 3.3% males and *n* = 4.4% females), with a nonsignificant value of *p* = 0.67 (Figure 6).

**Figure 7:**
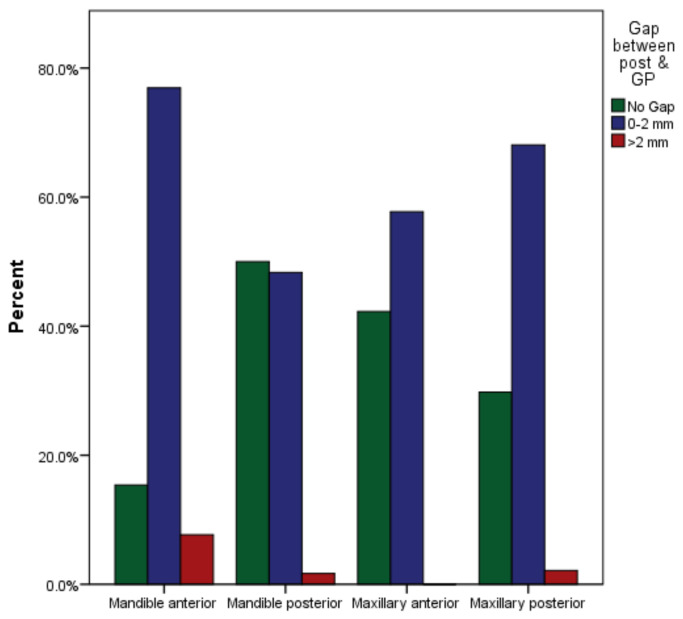
Gap between post and remaining gutta-percha in maxillary and mandibular anterior and posterior arches.

The maxillary anterior arch contained 97 endodontically treated teeth with post and core, 41 (42%) without a gap between the cemented post and gutta-percha, and 56 (58%) with >0 to 2 mm of gap between the cemented post and gutta-percha (Table 5). However, no teeth had more than 2 mm gap between cemented post and gutta-percha. Fourteen (30%) teeth in the 47 maxillary posteriors were cemented without gaps between the cemented post and the gutta-percha. A total of 32 (68%) teeth were restored with a gap between the cemented post and the remaining gutta perch of more than 0 to 2 mm, and there was one (2%) tooth that had a gap of more than 2 mm between the cemented post and the remaining gutta perch (Figure 7). There were a total of 13 teeth restored in the anterior region of the mandible, with two (15%) teeth being restored without any gap between the cemented post and the remaining gutta-percha, 10 (77%) teeth receiving a gap between 0 and 2 mm, and one (8%) tooth receiving a gap greater than 2 mm. Thirty (50%) of 60 endodontically treated mandibular posterior arch teeth were restored without gaps. A total of 29 (48%) teeth were restored with a gap >0 to 2 mm, and one (2%) tooth was restored with a gap >2 mm. With a *p* value of 0.048, the outcome of the study was highly significant.

**Figure 8:**
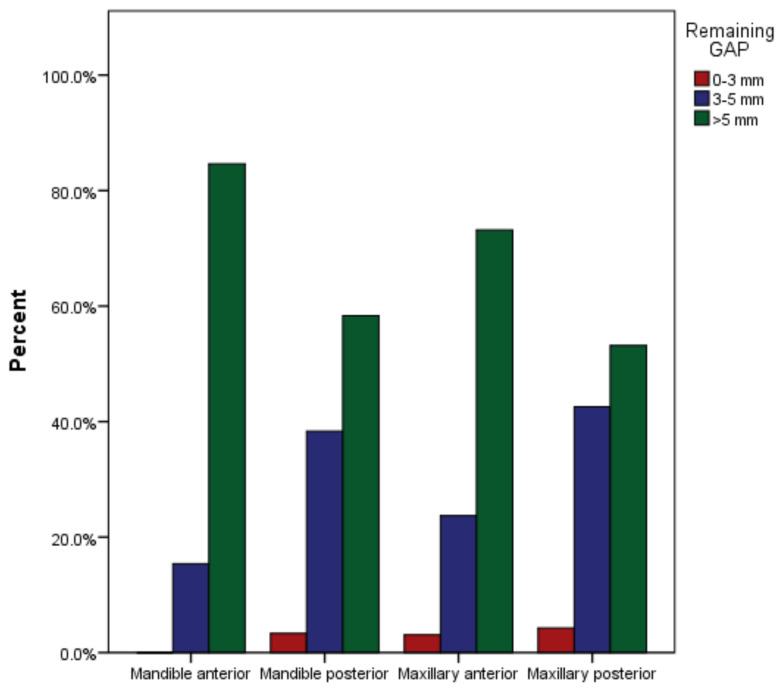
Remaining gutta-percha in maxillary and mandibular anterior and posterior arches after post cementation.

A total of 97 endodontically treated teeth with post and core in the maxillary anterior arch 71 (73%) had more than 5 mm of gutta-percha remaining, 23 (24%) had 3 to 5 mm. There were three (3%) teeth with less than 3 mm of gutta-percha left. Twenty-five (53%) of the 47 posterior maxillary teeth had more than 5 mm of gutta-percha left. Despite this, there were 20 (43%) teeth with 3 to 5 mm of remaining gutta-percha, and two (4%) teeth with less than 3 mm. The mandibular anterior region had 13 teeth restored, 11 (85%) with more than 5 mm of remaining gutta-percha, and two (15%) with 3 to 5 mm (Table 6). On the other hand, no anterior mandibular teeth with less than 3 mm of gutta-percha remaining on them were observed. The posterior mandibular arch was treated with 60 post and core teeth, of which 35 (58%) were restored with more than 5 mm of gutta-percha remaining. A total of 23 (38% of the teeth) were restored with 3 to 5 mm of gutta-percha. Only two (3%) teeth had less than 3 mm of gutta-percha remaining. The *p* value of 0.151 indicates a highly significant outcome (Figure 8)

## 4. Discussion

The prognosis of an endodontically treated tooth is directly impacted by the quality of the restorative treatment performed after root canal treatment [17,18]. Root canal-filled teeth are often restored using a post and core because of the loss of structural integrity as a result of removal of extensive caries and existing restorations during endodontic cavity preparation, which results in a lack of sufficient hard tissue support for a permanent coronal restoration. An adequate post length has been recommended by several authors in the literature to support the core. Goodacre and Spolnik recommended that the length of a post should be equal to three-quarters of the length of the root canal, if possible, or at least equal to the length of the crown [19]. To maintain an adequate seal, 4 to 5 mm of gutta-percha should remain apically. Using a retrospective study, Sorensen and Martinoff reported a 97% success rate if the post length is at least equaled the crown height [20]. In a study conducted by Neagley, an 8 mm post is the minimum length that should be used [21]. The apical portion of the root should have at least 3–5 mm of gutta-percha to maintain an adequate apical seal [22,23,24]. In a recent study by Abramovitz et al., it was evidenced that 3 mm of gutta-percha provided an unreliable apical seal. Therefore, 4 to 5 mm of remaining gutta-percha was recommended. It was stated that not only the length of the remaining root canal filling, but also the adhesion between the post and the root canal dentin, played a key role in coronal microleakage. It has also been pointed out that the seal of both post and cores had to be improved for the prevention of recontamination [25]. It has been reported that the coronal gutta-percha may be contaminated with bacteria in a matter of days if the coronal gutta-percha is exposed to bacterial contamination [26,27]. Bacterial by-products and endotoxins can penetrate to the apex in a very short period of time [5]. It is evident that the sealing ability of remaining root canal filling, the needless weakening of the root, and the timing of post space preparation all play a significant role in the success of teeth restored with posts.

In a recent study by Joshua Moshonov, it was found that clinical outcomes were significantly adverse when there was a gap of more than 2 mm between the gutta-percha and the cemented post. There was only a 29% success rate with post-and-core restorations for endodontically treated teeth [14]. This may be caused by the microleakage of saliva, dye, and anaerobic bacteria following post and core treatment. A favorable outcome was obtained in 53% of cases, even when the gap was under 2 mm. After 5 years, the tooth restored with no gap between the post and gutta-percha showed an 83% favorable outcome. According to the present study, 40% of the apical portion of posts were in contact with gutta-percha. A gap of less than 2 mm was found in 59% of the posts. The gap between the post end and remaining gutta-percha was greater than 2 mm in only 1% of the total cases. A similar study by Ozkurt found that 207 teeth had no gap between the post restoration and the remaining root canal filling [28]. A total of 135 (65%) teeth displayed healthy periapical tissue and 72 (35%) displayed signs of apical periodontitis. 81 teeth had a gap between the remaining root canal filling and the post restoration. Among these teeth, 69 (85%) had peri-apical pathosis, but only 12 (15%) had healthy periapical tissue. 

Kvist et al. stated that 3 mm residual apical gutta-percha filling after preparation of the post space is associated with a higher incidence of periapical radiolucency than roots with longer residual fillings [29]. According to De Cleen A minimum of 3 mm of gutta-percha should remain in the root canal [30]. The teeth with a minimum of 5 mm of apical root canal fillings were considered as successful endodontic treatment. In the present study, 65% of the endodontically treated teeth with post and core had more than 5 mm of remaining gutta-percha. There were fewer than 3% of cases with less than 3 mm of residual remaining gutta-percha. It was demonstrated that 3 mm of gutta-percha does not provide a reliable apical seal, so 4 to 5 mm of remaining gutta-percha should be present to maintain the apical seal [25]. As per traditional teachings, it is recommended that a minimum of 3–5 mm of gutta-percha must remain in the apical portion of the root to ensure that the seal is maintained. A 5 mm root canal filling has been accepted for years for maintaining the seal similar to an intact root canal filling. 

During the restoration of grossly decayed teeth with post and core, preoperative radiograph plays a very important role in identifying the peri-radicular status of the tooth, existence of the periapical lesion, and also the anatomical morphology of the root canals [31]. Any morphological variation in the root canal, such as dilaceration, curved canal or multiple canals are identified prior to the post and core treatment, if neglected results in failure of endodontic treatment. Post and core treatment procedure for restoring a grossly decayed teeth after root canal treatment is complex, and require the development of a high level of skill. It is, therefore, essential that undergraduate dental students are trained accordingly. The preclinical training program in Fixed Prosthodontics includes the theory and practical training for post and core treatment on endodontically treated teeth. This preclinical training has been demonstrated to play an important role which facilitates the smooth transition to the clinical procedures [32]. A positive correlation has been established between preclinical grades and clinical grades in fixed prosthodontics [33]. During the preclinical training program in College of Dentistry, Jazan University, Saudi Arabia, the undergraduate students are trained to perform the post and core treatment on artificial teeth. The training on artificial teeth provides an appropriate and realistic simulation. Students are also trained to perform the root canal treatment in the same teeth prior to the post and core treatment. The undergraduate students had properly understood the anatomy of the root canal. There is no doubt that the post space preparation should not compromise the integrity of the remaining root canal filling or the morphology of the canal. A combination of removing gutta-percha using heat pluggers followed by the post drill was the safest method, was used to train the undergraduate students. The underperforming students had repeatable trainings to learn the procedure. The results of this study explain the favorable radiographic outcome of the post and core treatment procedures on patients after the proper preclinical laboratory training. The study was also planned to identify the treatment outcome performed by male and female undergraduate students separately, since the educational institutions inside the Kingdom of Saudi Arabia have separate male and female training sections, where male undergraduate students can treat only male patients and female undergraduate students can treat only female patients. The inclusion of gender differences might reveal the skill deficiency between the male and female undergraduate students and may necessitate more training for the identified gender. Despite the benefits of CBCT over intra oral peri-apical radiographs, its application is limited by the high radiation dose [34,35]. The present study also included metal cast posts which may have resulted in the inaccurate measurements because the presence of metal causes a greater loss of data in the image reconstruction process and result with the formation of beam hardening artefacts [36]. Despite the three-dimensional nature of reconstructed CBCT images, the poor resolution of CBCT and artefacts caused by gutta-percha could also lead to inaccuracy [37]. The present retrospective study focused on the treatment outcome of the post- and core-treated teeth by undergraduate students in terms of the gap between post end and remaining gutta-percha. Students were trained to take the periapical radiographs at each stage of post and core treatment in preclinical training and then perform the same procedure in the clinical training. Taking a CT scan was not practical in preclinical and clinical trainings for undergraduate students. For an accurate anatomy reproduction, paralleling technique was used with the image receptor kept parallel to the longitudinal tooth axis.

As a limitation of this study, there is a need for future studies to compare the clinical outcomes of gaps between posts and remaining gutta-percha performed by the undergraduate students at different academic levels in an institution.

## 5. Conclusions

Most of the endodontically treated teeth with post and core (65%) had more than 5 mm of remaining gutta-percha after post and core treatment performed by the undergraduate students. Fewer than 3% of the cases had less than 3 mm of remaining gutta-percha. In terms of the gap between the post end and the remaining gutta-percha, the quality of the endodontically treated teeth by final year students is clinically acceptable. Almost 99% of the teeth had less than 2 mm gap between the post end and the remaining gutta-percha. Undergraduate students receive sufficient preclinical laboratory training to perform post and core treatment on endodontically treated teeth.

## Figures and Tables

**Table 1:** Gap between post and remaining gutta-percha in maxillary and mandibular arches.

Arch	Gap between Post & Remaining Gutta-Percha			Total	*p* Value
	No Gap	>0 to 2 mm	>2 mm		
Maxilla	54 (38%)	88 (62%)	1 (0.7%)	143 (100%)	0.268
Mandible	33 (45%)	39 (53%)	2 (2.7%)	74 (100%)	
Total	87 (40%)	127 (59%)	3 (1.4%)	217 (100%)	

**Table 2:** Gap between post and remaining gutta-percha among male and female students.

Gender	Gap between Post & Remaining Gutta-Percha			Total	*p* Value
	No Gap	>0 to 2 mm	>2 mm		
Male	63 (60%)	42 (40%)	0 (0%)	105 (100%)	*p* < 0.001
Female	24 (21%)	85 (76%)	3 (3%)	112 (100%)	
Total	87 (40%)	127 (59%)	3 (1%)	217 (100%)	

**Table 3:** Remaining gutta-percha after post cementation among male and female students.

Gender	Remaining Gutta-Percha after Post Cementation			Total	*p* Value
	0–3 mm	3–5 mm	>5 mm		
Male	3 (3%)	34 (32%)	68 (65%)	105 (100%)	0.918
Female	4 (4%)	34 (30%)	74 (66%)	112 (100%)	
Total	7 (3%)	68 (31%)	142 (65%)	217 (100%)	

**Table 4:** Remaining gutta-percha after post cementation in maxillary and mandibular arches.

Arch	Remaining Gutta-Percha after Post Cementation			Total	*p* Value
	0–3 mm	3–5 mm	>5 mm		
Maxilla	5 (4%)	42 (29%)	96 (67%)	143 (100%)	0.671
Mandible	2 (3%)	26 (35%)	46 (62%)	74 (100%)	
Total	7 (3%)	68 (31%)	142 (65%)	217 (100%)	

**Table 5:** Gap between post and remaining gutta-percha in maxillary and mandibular anterior and posterior arches.

Arch	Gap between Post & Remaining Gutta-Percha			Total	*p* Value
	No Gap	>0 to 2 mm	>2 mm		
Maxillary anterior	41 (42%)	56 (58%)	0 (0%)	97 (100%)	0.048
Maxillary posterior	14 (30%)	32 (68%)	1 (2%)	47 (100%)	
Mandibular anterior	2 (15%)	10 (77%)	1 (8%)	13 (100%)	
Mandibular posterior	30 (50%)	29 (48%)	1 (2%)	60 (100%)	
Total	87 (40%)	127 (59%)	3 (1.4%)	217 (100%)	

**Table 6:** Remaining gutta-percha in maxillary and mandibular anterior and posterior arches after post cementation.

Arch	Remaining Gutta-Percha after Post Cementation			Total	*p* Value
	0–3 mm	3–5 mm	>5 mm		
Maxillary anterior	3 (3%)	23 (24%)	71 (73%)	97 (100%)	0.151
Maxillary posterior	2 (4%)	20 (43%)	25 (53%)	47 (100%)	
Mandibular anterior	0 (0%)	2 (15%)	11 (85%)	13 (100%)	
Mandibular posterior	2 (3%)	23 (38%)	35 (58%)	60 (100%)	
Total	7 (3%)	68 (31%)	142 (65%)	217 (100%)	

## Data Availability

The data that support the findings of this study are available from the corresponding author upon reasonable request.
